# Supplementary material for: Explainable deep learning for insights in El Niño and river flows
Source: Nat Commun. 2023 Jan 20;14:339. doi: 10.1038/s41467-023-35968-5 (PMC9860069; doi:10.1038/s41467-023-35968-5)
Supplement: Supplementary file 1 — Supplementary Information [file 41467_2023_35968_MOESM1_ESM.pdf]

a

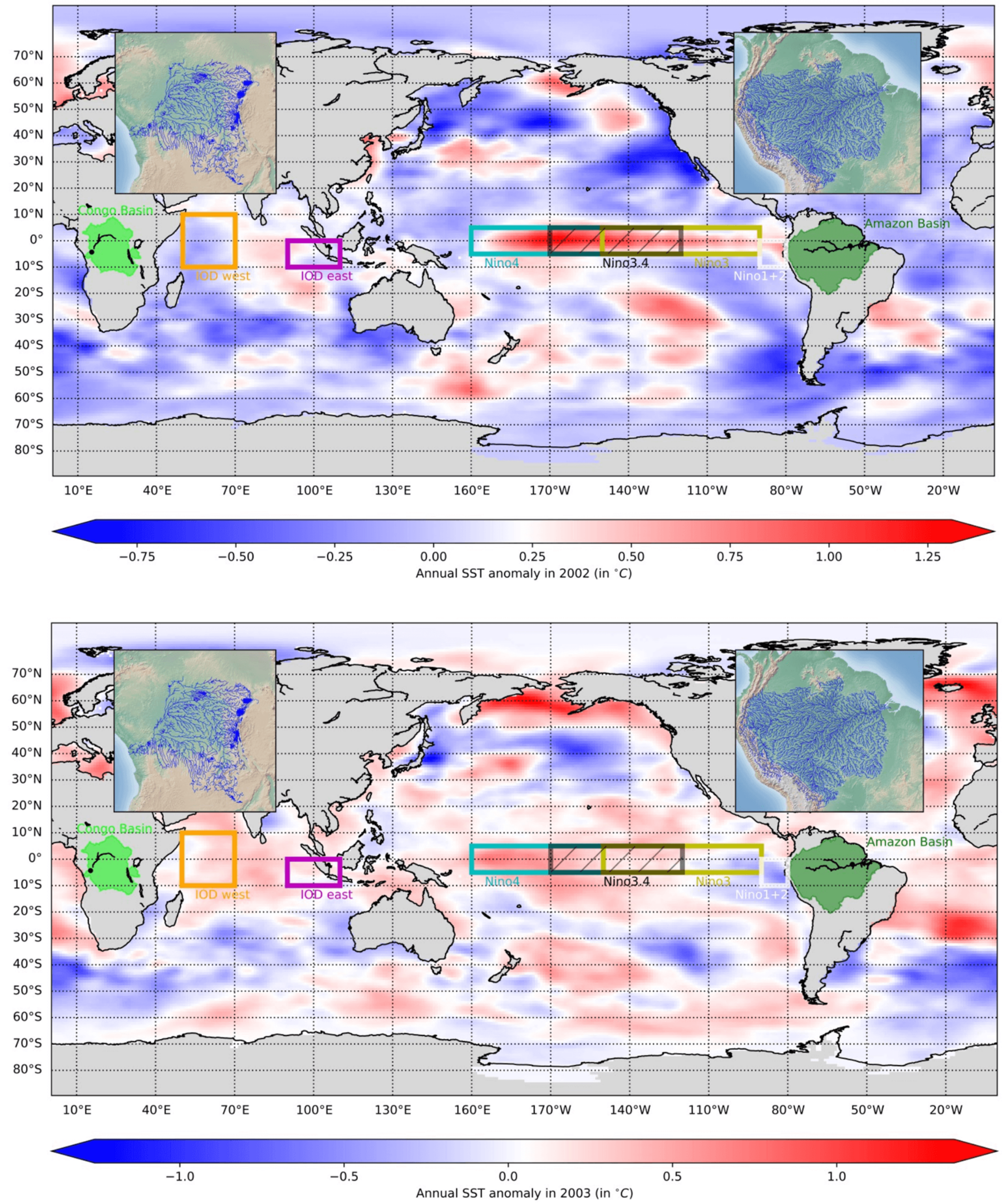

b

Figure S1. Global sea surface temperature anomalies in an El Niño year and a neutral year. a, The year 2002 qualified as an El Niño year because a warm anomaly of +0.5°C or greater persisted for a minimum of 5 consecutive months. Outside of the Niño 3.4 region, the global sea surface temperature anomalies in 2002 were largely negative. Warming of surface waters the eastern Pacific during El Niño events is associated with weakening of trade winds along the equator, bringing severe rainfall and drought to far-flung regions. **b**, Neutral states often correspond to transitions between El Niño and La Niña events.

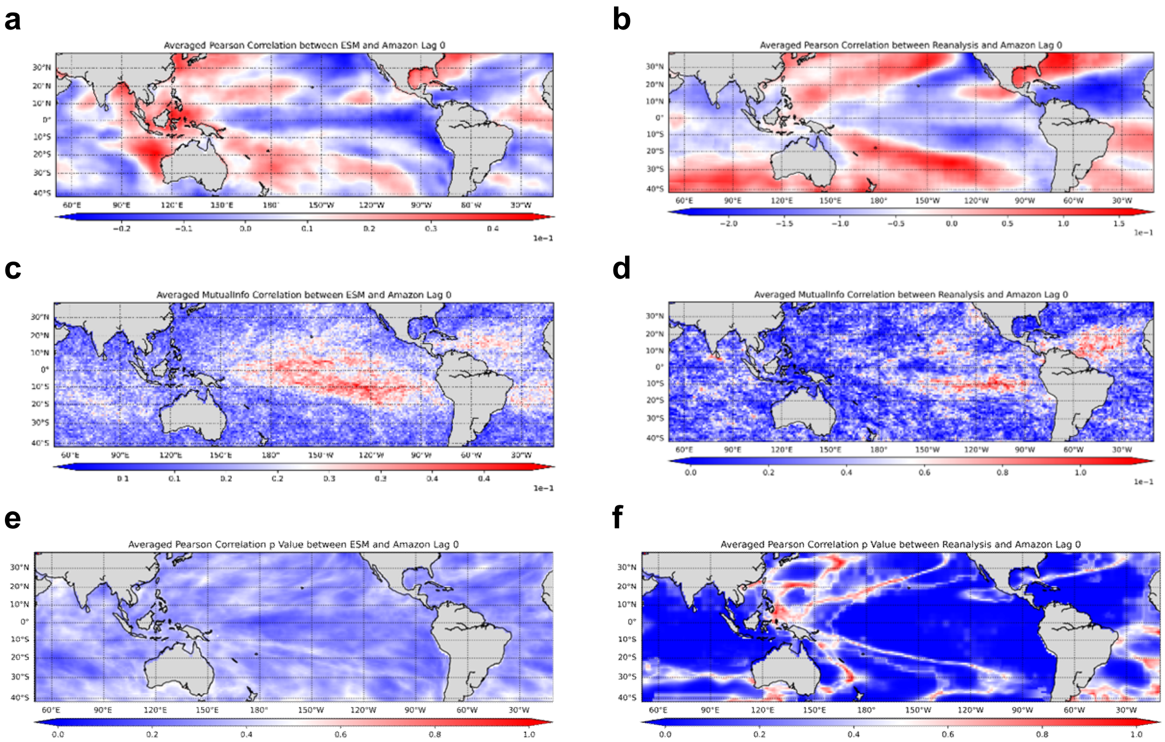

Figure S2. Linear and nonlinear dependence between Amazon River flow and global sea surface temperature. Time series of monthly Amazon River flow were analyzed with reference to sea surface temperature (SST) at each geographic location for a period of 672 months from January 1950 to December 2005. Pearson correlation of Amazon River flow with Earth System Model (ESM) SST (**a**) and reanalysis SST (**b**) Mutual information between Amazon River flow ESM SST (**c**) and reanalysis SST (**d**). The p-values of Pearson correlation of Amazon River flow with ESM SST (**e**) and reanalysis SST (**f**).

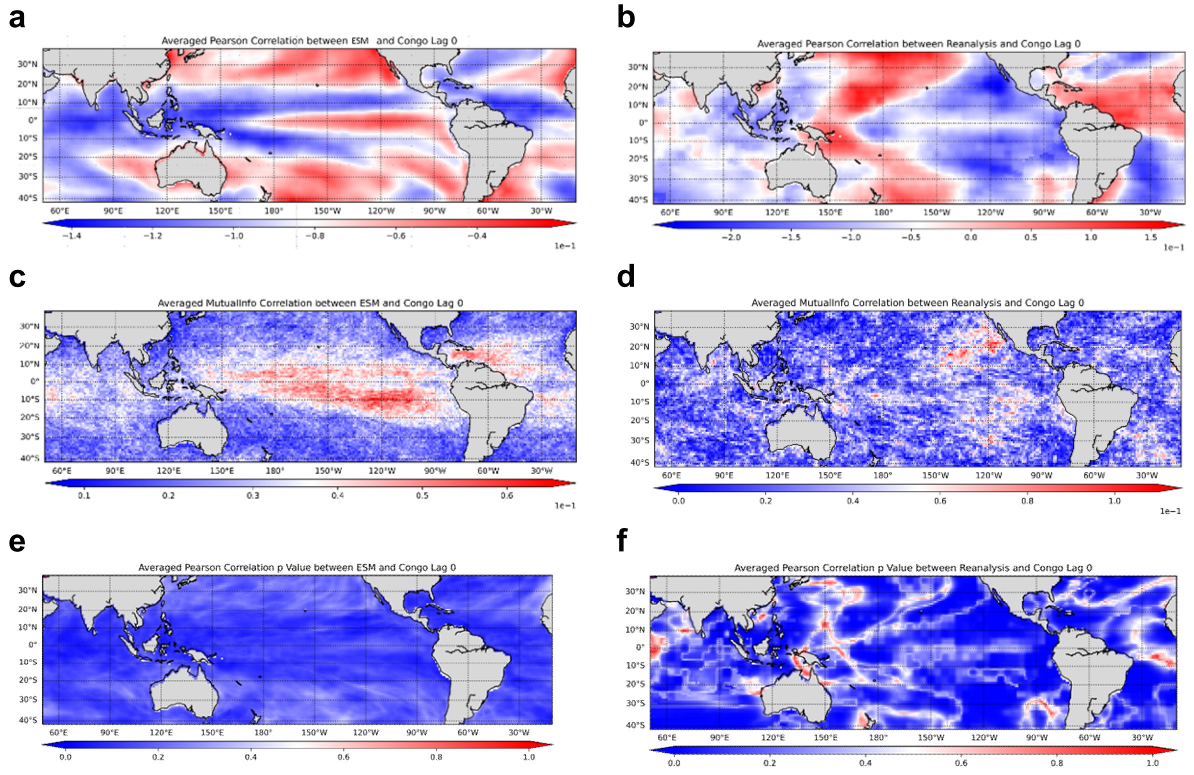

Figure S3. Linear and nonlinear dependence between Congo River flow and global sea surface temperature. Time series of monthly Congo River flow were analyzed with reference to sea surface temperature (SST) at each geographic location for a period of 672 months from January 1950 to December 2005. **a,b,** Pearson correlation of Congo River flow with Earth system model (ESM) SST (**a**) and reanalysis SST (**b**) Mutual information between Congo River flow ESM SST (**c**) and reanalysis SST (**d**). The p-values of Pearson correlation of Congo River flow with ESM SST (**e**) and reanalysis SST (**f**).

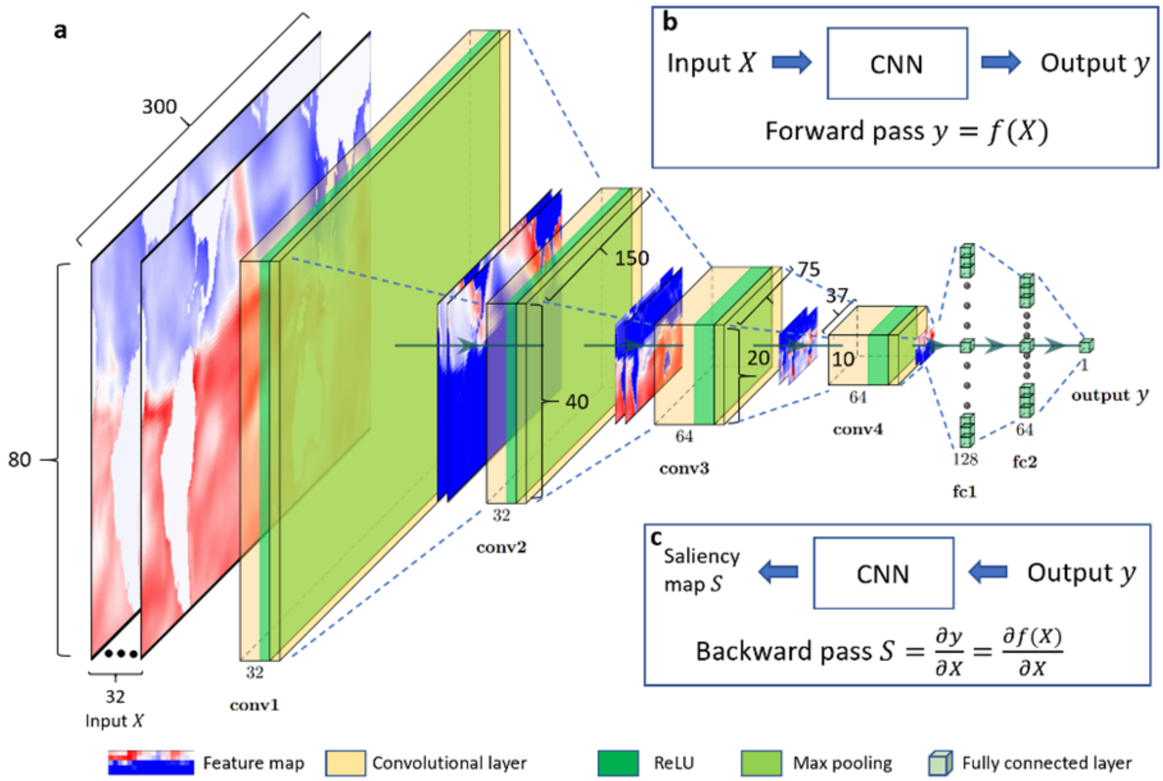

Figure S4. Architecture of convolutional neural network model used in this paper. a, Network architecture. There are 4 convolutional layers (conv1 - conv4) and 3 fully connected layers (fc1,2 and output). Each convolutional layer is followed by a ReLU activation (ReLU(x)=max(0,x)) and a max pooling layer. The image input size is 80×300×C with C = 1, 3 or 32 depending on the datasets. For the convolutional layers, the filter sizes are all 3×3 with stride 1 and padding 1; and the number of filter channel is 32, 32, 64 and 64, respectively. The pooling layers are 2D max pooling layers with size 2×2 and will reduce the feature maps to half size. The outputs of the fully connected layers are 1D vectors with length 128, 64 and 1, respectively. **b**, forward pass to input climate variable sea surface temperature as X and get prediction y. **c**, backward pass to calculate gradient of prediction y with respective to input X to get saliency maps.

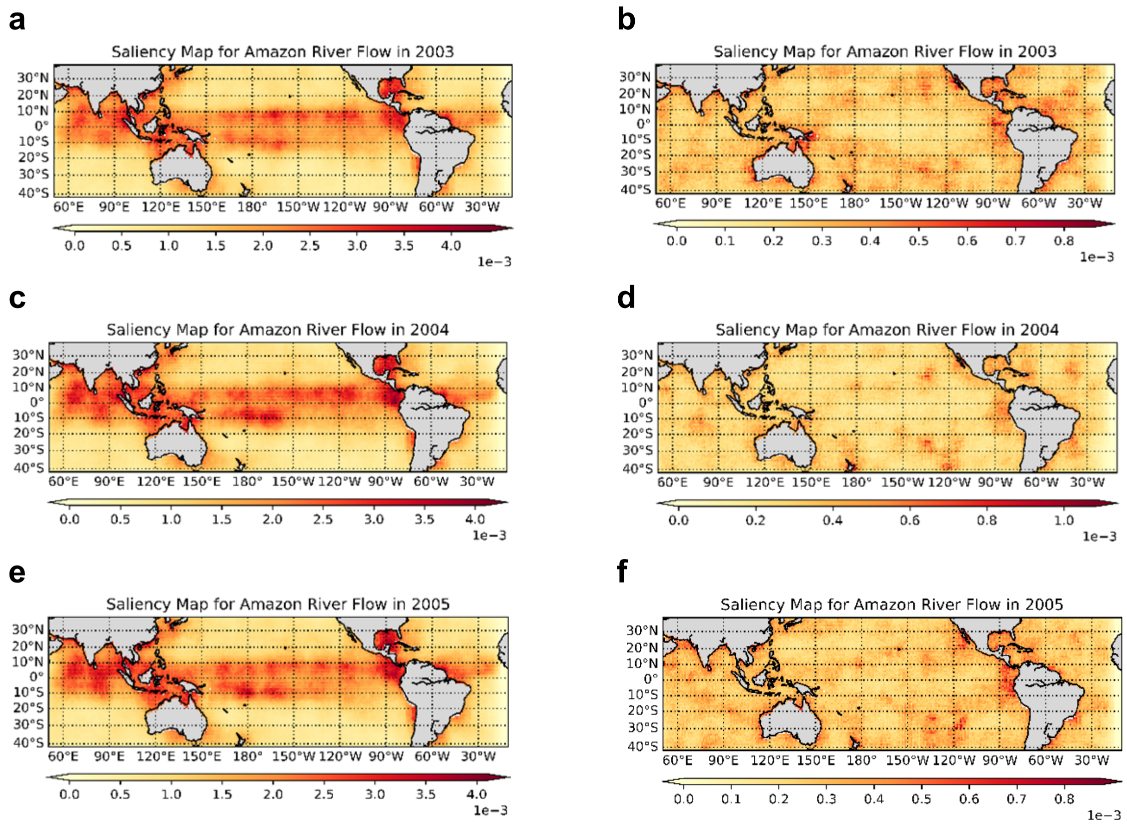

Figure S5. Yearly cyclical saliency maps highlight the relative contributions of geographic regions to predicting Amazon River flow. The yearly cyclical saliency maps are calculated as the mean of saliency maps of the 12 months in the corresponding year. **a,c,e** Salient Earth System Model (ESM) sea surface temperature (SST) regions for Amazon River flow prediction in the years 2003, 2004, and 2005 were clustered around the equator, but were not limited to the El Niño–Southern Oscillation region. **b,d,f** Salient reanalysis SST regions for Amazon River flow prediction were more diffused across latitudes than ESM regions.

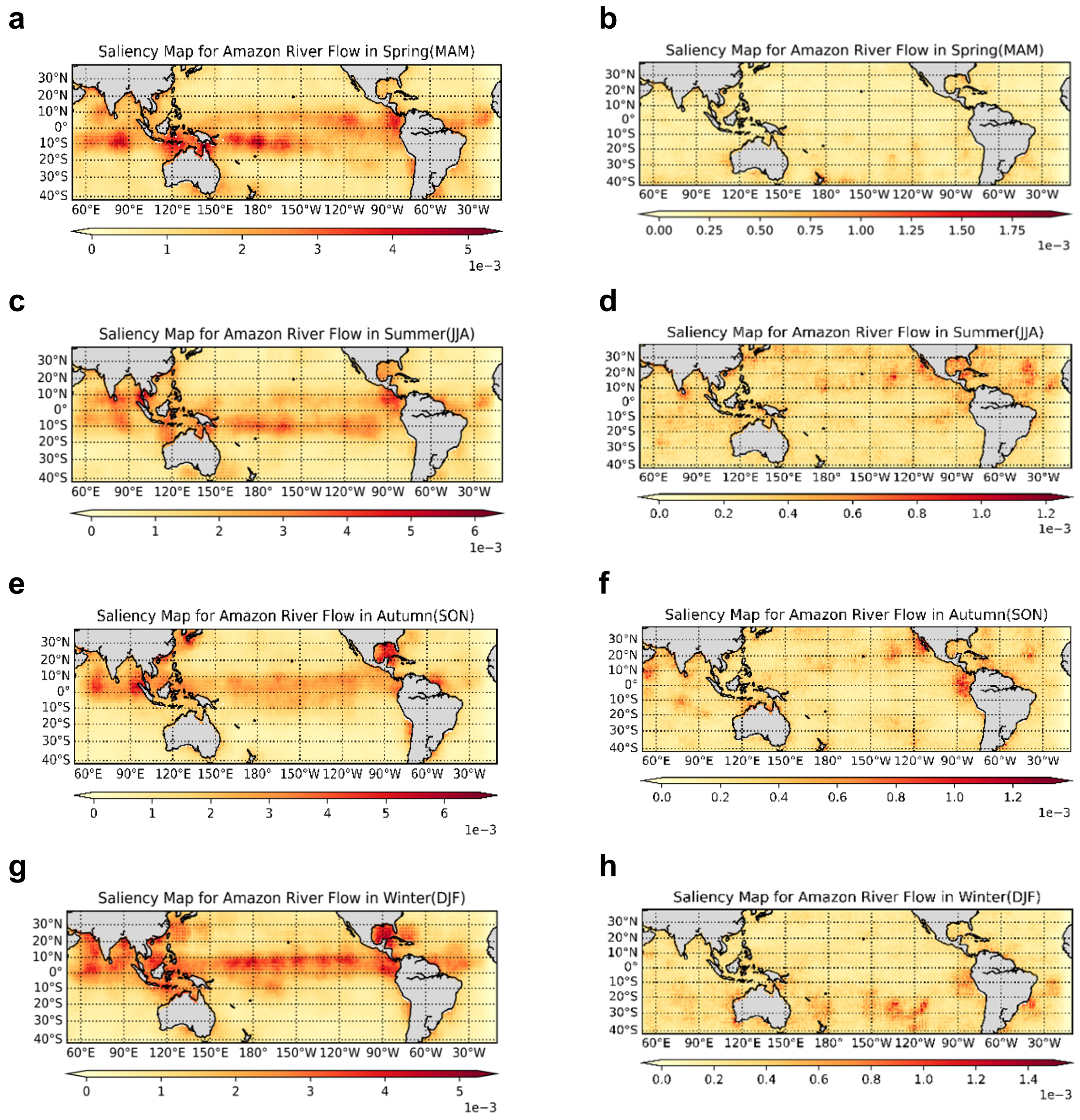

Figure S6. Seasonal cyclical saliency maps for show periodic changes in the relevant regions for predicting Amazon River flow. The seasonal cyclical saliency maps are calculated as the mean of saliency maps for different seasons in the Northern Hemisphere (spring: March, April, May; summer: June, July, August; autumn: September, October, November; winter: December, January and February). **a,c,e,g** Saliency maps based on Earth System Model sea surface temperature (SST). **b,d,f,h** Saliency maps based on reanalysis SST suggest more salient regions in the northern latitudes during Northern Hemisphere summer (June, July, August) and in the southern latitudes during Southern Hemisphere summer (December, January, February).

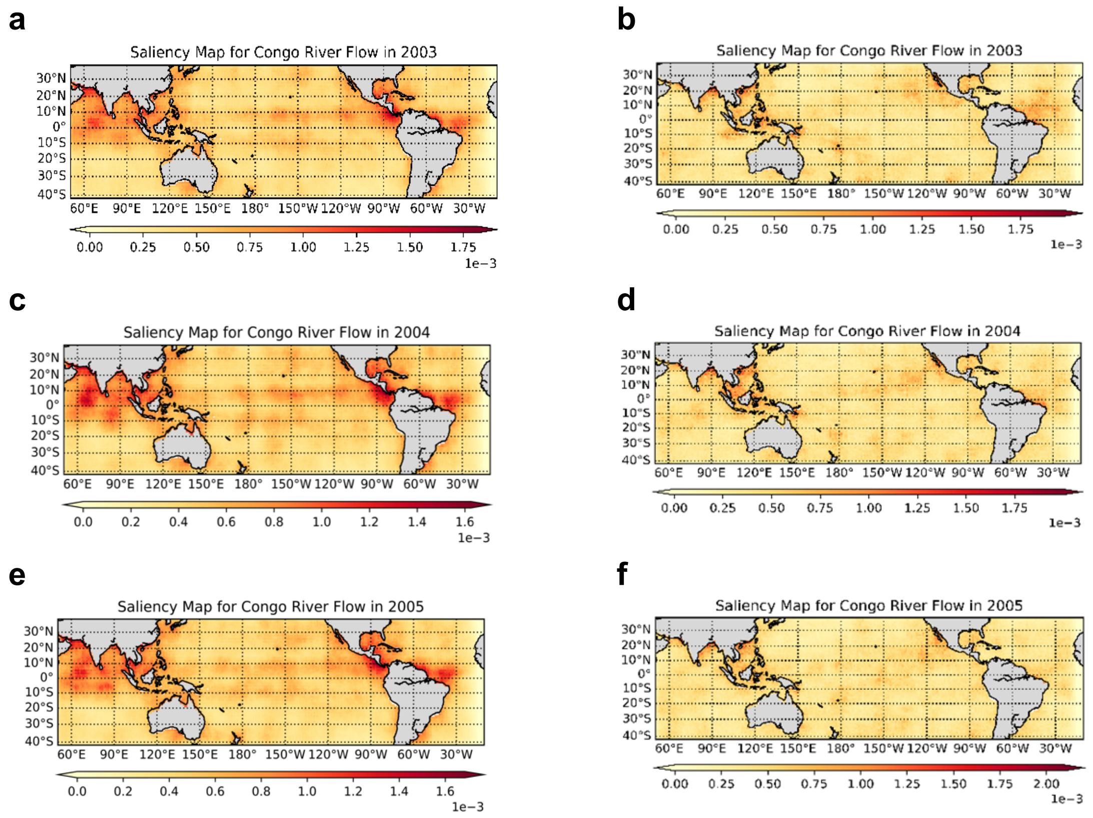

Figure S7. Yearly cyclical saliency maps highlight the relative contributions of geographic regions to predicting Congo River flow. The yearly cyclical saliency maps are calculated as the mean of saliency maps of the 12 months in the corresponding year. **a,c,e** Salient Earth System Model sea surface temperature (SST) regions for Congo River flow prediction in the years 2003, 2004, and 2005 were clustered around the equator, with Atlantic and Indian Ocean regions more salient than the El Niño–Southern Oscillation region. **b,d,f** Salient reanalysis SST regions for Congo River flow prediction were more diffused.

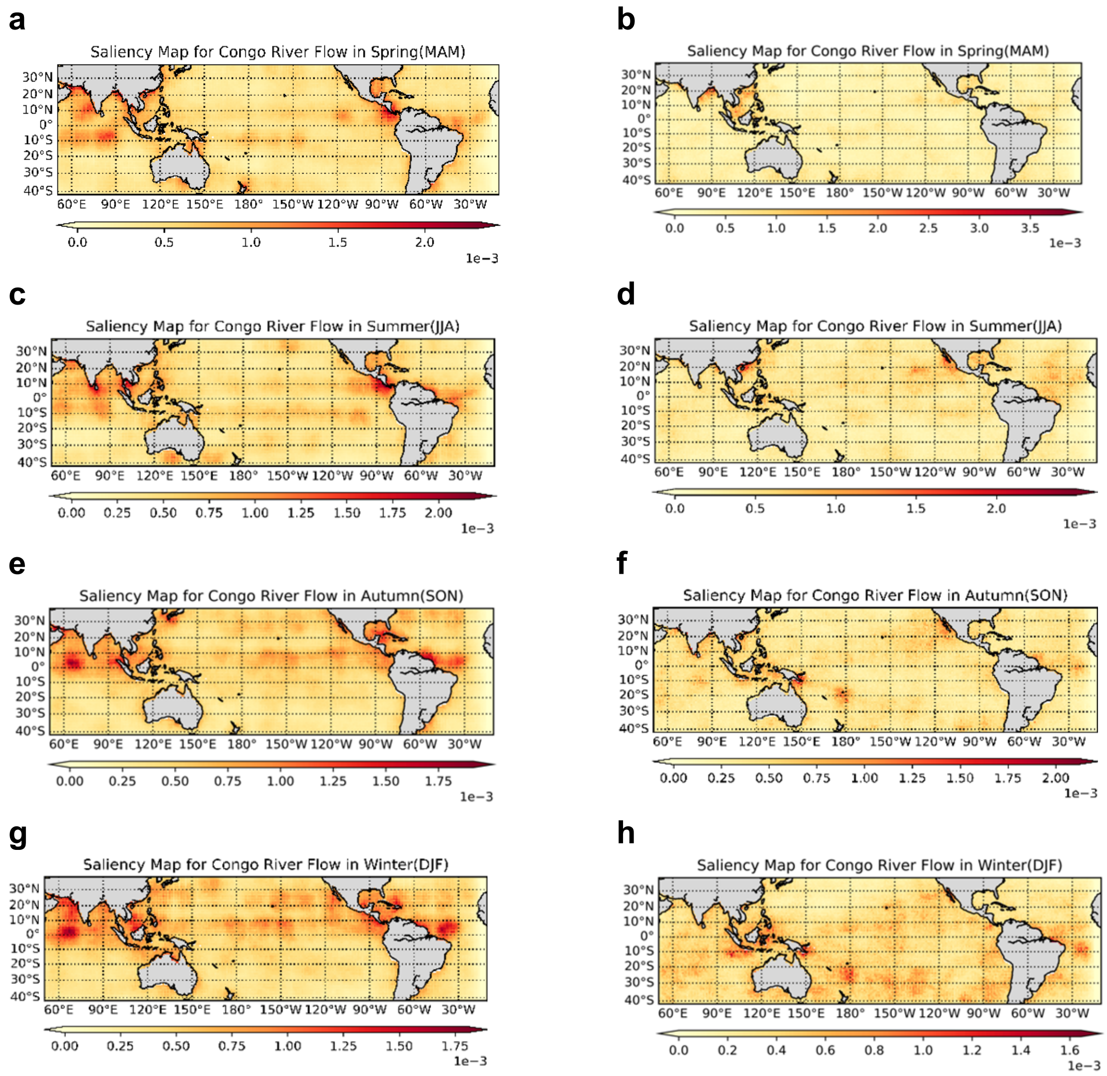

Figure S8. Seasonal cyclical saliency maps highlight the relative contributions of geographic regions to predicting Congo River flow. The seasonal cyclical saliency maps are calculated as the mean of saliency maps for different seasons. **a,c,e,g** Saliency maps based on Earth System Model (ESM) sea surface temperature (SST) reveal less salient information in the ENSO region than saliency maps for Amazon River prediction. **b,d,f,h** Saliency maps based on reanalysis SST reflect more diffused salient regions than ESM SST.

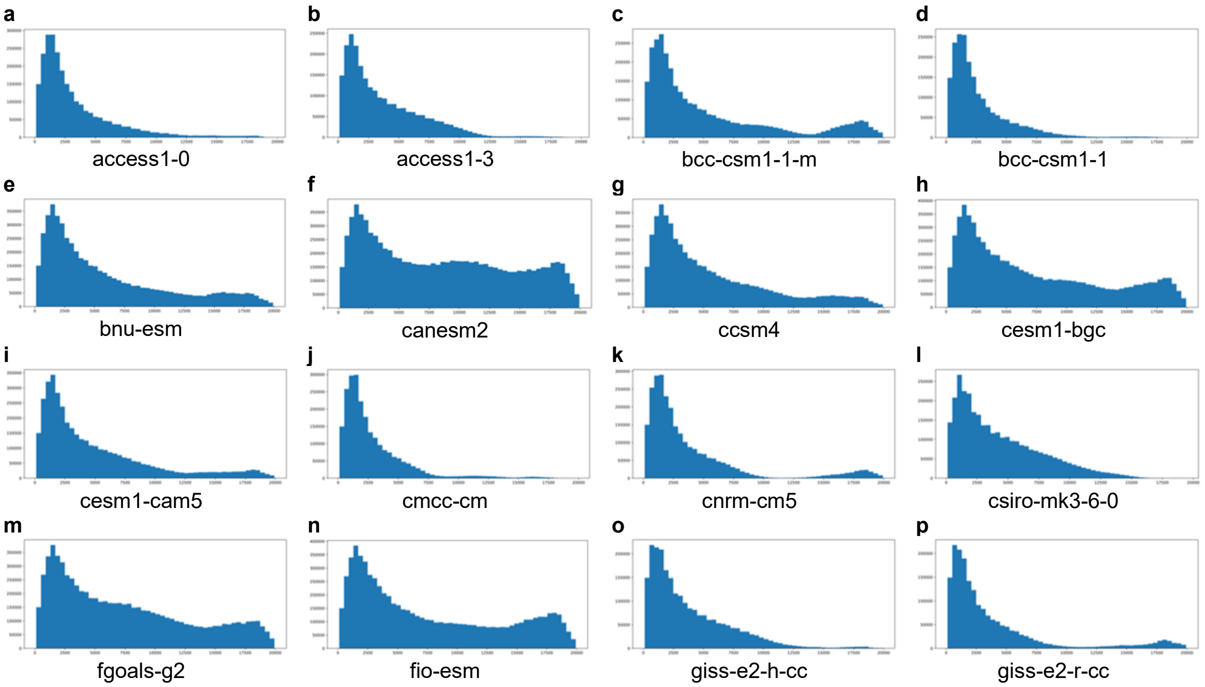

Figure S9. Diverse climate network topology is suggested by distributions of geographic distance between correlated sea surface temperature time series. Defining a connection as any pair of geographic locations where the Pearson correlation between the sea surface temperature (SST) time series is equal or greater than 0.5, the distribution of connection lengths is plotted for each Earth System Model (ESM) (**a-p**). Variation in the shape of distributions indicates dissimilar topology of climate networks among different ESMs, with substantial variation in the number of long range, or teleconnections. SST time series of 672 months spanning January 1950 to December 2005 were evaluated for locations in the area with latitude from 9.5°S to 9.5°N and longitude from 50.5°E to 349.5°E.

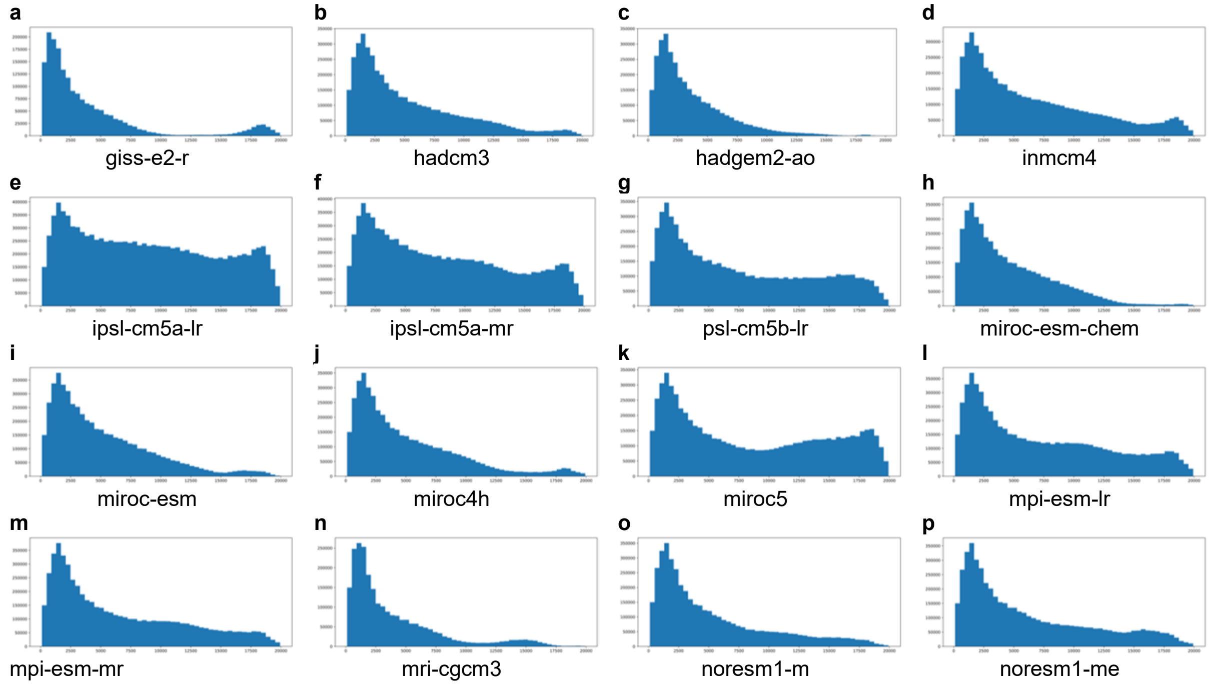

Figure S10. Diverse climate network topology is suggested by distributions of geographic distance between correlated sea surface temperature time series (continued). Defining a connection as any pair of geographic locations where the Pearson correlation between the sea surface temperature time series is equal or greater than 0.5, the distribution of connection lengths is plotted for each Earth System Model (**a-p**).

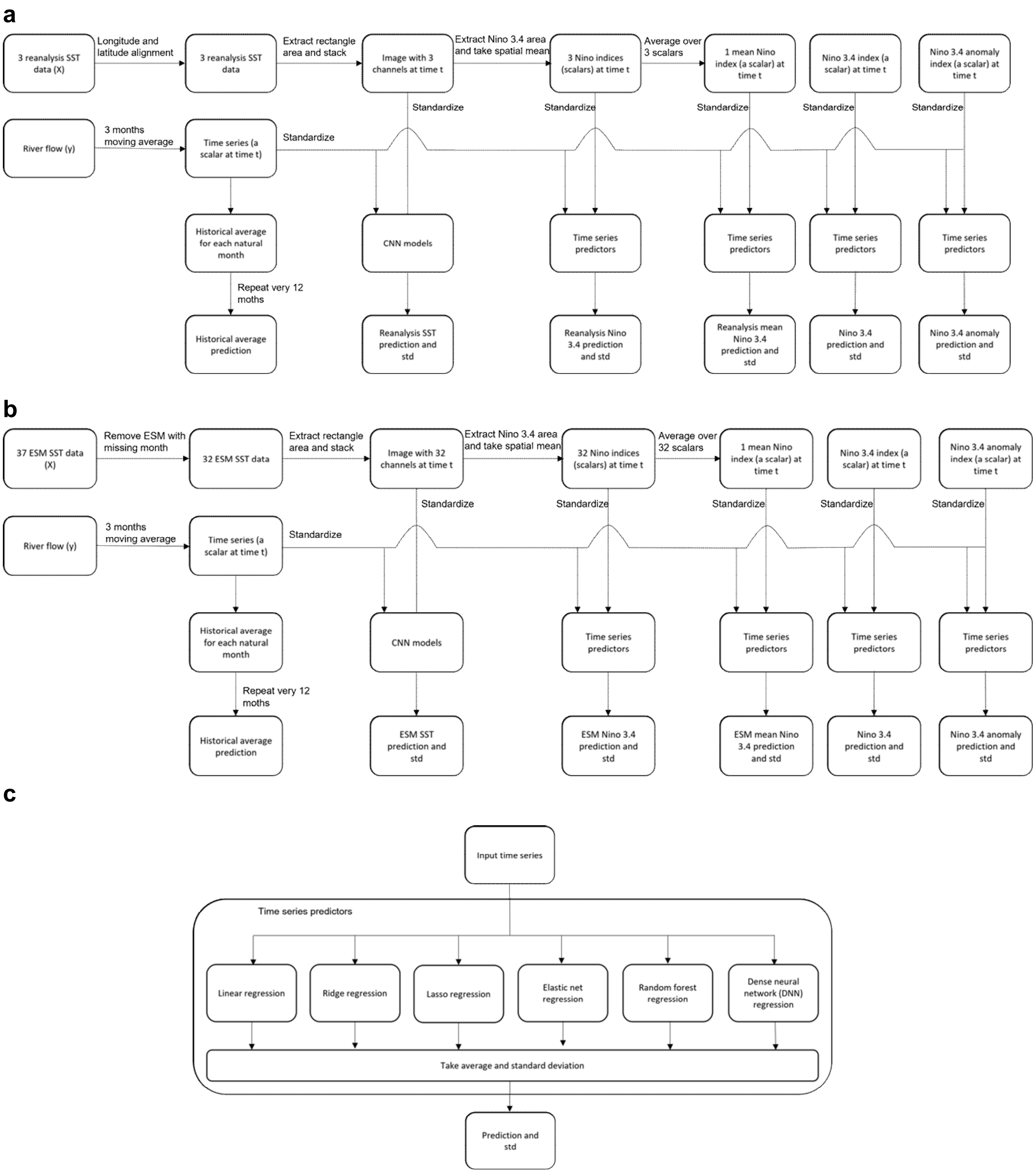

Figure S11. Flowcharts of dataset processing, river flow modeling, and evaluation of predictions. **a,** Reanalysis sea surface temperature (SST) is extracted at the Niño 3.4 region and subjected to various levels of spatial aggregation and averaging for use as inputs to machine learning (ML) models. Standardized river flow is used to generate a baseline prediction used on climatological mean, as well as a label for the ML models. **b**, Similar steps are applied to SST simulations from 32 Earth System Models. **c**, The ensembling method to generate probabilistic river flow predictions uses a suite of methods to produce a predictions, from which a mean value and predictive variance are calculated.

Table S1. Earth System Models used in the experiments. All models were obtained from the Coupled Model Intercomparison Project Phase 5.

Index	Name	Index	Name	Index	Name	Index	Name
0	access1-0	8	cesm1-cam5	16	giss-e2-r	24	miroc-esm
1	access1-3	9	cmcc-cm	17	hadcm3	25	miroc4h
2	bcc-csm1-1-m	10	cnrm-cm5	18	hadgem2-ao	26	miroc5
3	bcc-csm1-1	11	csiro-mk3-6-0	19	inmcm4	27	mpi-esm-lr
4	bnu-esm	12	fgoals-g2	20	ipsl-cm5a-lr	28	mpi-esm-mr
5	canesm2	13	fio-esm	21	ipsl-cm5a-mr	29	mri-cgcm3
6	ccsm4	14	giss-e2-h-cc	22	ipsl-cm5b-lr	30	noresm1-m
7	cesm1-bgc	15	giss-e2-r-cc	23	miroc-esm-chem	31	noresm1-me

Table S2. RMSE for predicting Amazon and Congo River flow using Niño 3.4 region sea surface temperature and larger area (5°S-5°N, 170°W-120°W) SST. Values with red bold and bold font indicate the best and second-best results on each task, respectively. Values marked with an ∗ indicate the best results when using Niño 3.4 region sea surface temperature as the predictor.

Method		Linear, lasso, ridge, elastic net, random forest and DNN regression						Historical average	CNN	
Predictor type		Niño 3.4 average SST (C-dimensional time series, C=1, 3 or 32)					Niño 3.4 index	Climatological	SST (2-dimensional images)	
Predictor source		ESM mean	ESM	HadISST1	Reanalysis mean	Reanalysis	HadISST1	Historical mean	ESM	Reanalysis
Amazon	Linear	1.051	0.508*	0.925	0.919	0.925	0.763			
	Ridge	1.047	0.499*	0.925	0.920	0.925	0.763			
	Lasso	1.050	0.576*	0.925	0.919	0.925	0.763			
	Elastic net	1.028	0.469*	0.925	0.922	0.925	0.762	**0.294**	**0.287**	0.301
	Random forest	1.216	0.518*	0.958	1.002	0.943	0.670			
	DNN	1.002	0.516*	0.954	0.929	0.954	0.770			
	Ensemble	1.049	0.461*	0.931	0.925	0.928	0.950			
Congo	Linear	1.012	0.802	0.996*	0.976	0.987	0.722*			
	Ridge	1.012	0.784	0.996*	0.977	0.985	0.722*			
	Lasso	1.022	0.848	0.996	0.976	1.000	0.723*			
	Elastic net	1.022	0.799	0.996	0.976	0.993	0.723*	**0.476**	0.779	**0.462**
	Random forest	1.240	0.804*	1.074	1.089	1.108	0.816			
	DNN	1.025	0.711*	1.000	0.980	0.980	0.728			
	Ensemble	1.043	0.750	1.005	0.992	0.999	1.027			

Table S3. Dependence between Niño indices and river flows. The relationships between river flows and sea surface temperature (SST) are analyzed using Pearson correlation, a measure of linear dependence, and mutual information, a nonlinear measure of the information content shared between two random variables. For the Niño 3.4 region SST, Earth System Model (ESM) mean is the average SST of 31 ESMs, HadISST1 is a single reanalysis model, and Reanalysis mean is the average SST of 3 reanalysis models.

Index		ESM mean	HadISST1	Reanalysis mean
Amazon	Pearson correlation	-0.0561	-0.191	-0.146
	Mutual information	0.049	0.077	0.077
Congo	Pearson correlation	-0.195	-0.024	-0.091
	Mutual information	0.058	0.008	0.097

Table S4. Different metrics for three prediction results for Amazon River. For Pearson, Spearman and Kendall's Tau correlation, the values in the parenthesis are the correlation and p-value, respectively. For the seasonal root mean squared error (RMSE), the values in the parenthesis are (MAM, JJA, SON, DJF). For the yearly RMSE, the values in the parenthesis are RMSE for the years 2003, 2004 and 2005, respectively. For the extreme RMSE, the values in the parenthesis are RMSE for predictions whose absolute values are within and outside 2 standard deviations from the mean, respectively. For above/below RMSE, the values in the parenthesis are RMSE for predictions whose values are above and below the mean (0), respectively. For El Niño–Southern Oscillation RMSE, the values in the parenthesis are RMSE for warm, cool and neutral months, respectively.

Metric / Method	Historical average	ESM+CNN	Reanalysis+CNN
Pearson correlation	(0.9637, 4.426e-21)	(0.967, 9.217e-22)	(0.9451, 4.397e-18)
Spearman correlation	(0.9504, 8.029e-19)	(0.9681, 5.171e-22)	(0.9284, 3.475e-16)
Kendall's tau correlation	(0.8402, 2.067e-12)	(0.8635, 1.265e-13)	(0.7778, 2.485e-11)
Mutual information	1.2425	1.2292	1.1371
Seasonal RMSE	(0.1695, 0.2220, 0.2407, 0.4576)	(0.2830, 0.2167, 0.1109, 0.3759)	(0.1941, 0.1722, 0.2789, 0.4956)
Yearly RMSE	(0.1728, 0.2338, 0.4177)	(0.2904, 0.2362, 0.2652)	(0.2382, 0.1717, 0.4548)
Extreme RMSE	(0.2622, 0.3145)	(0.2773, 0.2545)	(0.3339, 0.2998)
Above/below RMSE	(0.2815, 0.3057)	(0.2058, 0.3083)	(0.2961, 0.3281)
ENSO RMSE	(0.2040, 0.5991, 0.2868)	(0.2794, 0.2333, 0.2611)	(0.1820, 0.6420, 0.3136)
MAE	0.2215	0.2078	0.2382
Nash–Sutcliffe coefficient	0.9053	0.9231	0.893

Table S5. **Different metrics for three prediction results for Congo River.** As in table S4, for Pearson, Spearman and Kendall's Tau correlation, the values in the parenthesis are the correlation and p-value, respectively. For the seasonal root mean squared error (RMSE), the values in the parenthesis are (MAM, JJA, SON, DJF). For the yearly RMSE, the values in the parenthesis are RMSE for the year 2003, 2004 and 2005, respectively. For the extreme RMSE, the values in the parenthesis are RMSE for predictions whose absolution values are within and outside 2 standard deviations, respectively. For above/below RMSE, the values in the parenthesis are RMSE for predictions whose values are above and below the mean (0), respectively. For El Niño–Southern Oscillation (ENSO) RMSE, the values in the parenthesis are RMSE for warm, cool and neutral months, respectively.

Metric / Method	Historical average	ESM+CNN	Reanalysis+CNN
Pearson correlation	(0.909, 1.792e-14)	(0.8726, 4.051e-12)	(0.8994, 9.031e-14)
Spearman correlation	(0.8544, 3.397e-11)	(0.8682, 6.962e-12)	(0.86, 1.826e-11)
Kendall's tau correlation	(0.6866, 9.2312e-09)	(0.6921, 2.873e-09)	(0.673, 7.683e-09)
Mutual information	0.7301	0.5913	0.7445
Seasonal RMSE	(0.4908, 0.4468, 0.5885, 0.3440)	(0.9007, 0.4162, 0.3427, 0.5716)	(0.5257, 0.4639, 0.5788, 0.3400)
Yearly RMSE	(0.4866, 0.5178, 0.4171)	(0.8148, 0.4889, 0.4105)	(0.5434, 0.4873, 0.4172)
Extreme RMSE	(0.4122, 0.4874)	(0.4217, 0.6528)	(0.4417, 0.4971)
Above/below RMSE	(0.4612, 0.4858)	(0.8170, 0.4494)	(0.4985, 0.4758)
ENSO RMSE	(0.6421, 0.3819, 0.3943)	(0.7951, 0.5438, 0.4976)	(0.6423, 0.2926, 0.4176)
MAE	0.3879	0.4765	0.3895
Nash–Sutcliffe coefficient	0.7783	0.65	0.7691
